# Mothers stick together: how the death of an infant affects female social relationships in a group of wild bonobos (*Pan paniscus*)

**DOI:** 10.1007/s10329-022-00986-2

**Published:** 2022-04-18

**Authors:** Leveda Cheng, Amber Shaw, Martin Surbeck

**Affiliations:** 1 Department of Human Evolutionary Biology, Harvard University, 11 Divinity Avenue, Cambridge, MA 02138 USA; 2 Max Planck Institute for Evolutionary Anthropology, Deutscher Platz 6, 04103 Leipzig, Germany

**Keywords:** *Pan paniscus*, Female sociality, Social bonds, Proximity, Grooming, Mother-bonding hypothesis

## Abstract

Sociality is widespread among group-living primates and is beneficial in many ways. Sociality amongst female bonobos (*Pan paniscus*) has been proposed to have evolved as a female counterstrategy to male infanticide and sexual coercion. In male-philopatric bonobo societies, females mostly form relationships with unrelated females. Among these social relationships, it has been proposed that females with infants (also referred to as mothers) tend to have strong relationships with each other (mother-bonding hypothesis). In this paper, we use the case of an infant death in a group of wild bonobos in the Kokolopori Bonobo Reserve, Democratic Republic of Congo, to test this hypothesis. By using dyadic sociality indices for grooming, proximity, and aggression, we investigated whether the infant death influenced dyadic relationships the mother had with other group members. Before the infant death, grooming index (GI) and proximity index (PI) scores were the highest between the focal mother and another mother. After the death, the relationship of this mother dyad weakened, as indicated by lower GI and PI scores, whereas the relationship of another mother dyad became stronger. Aggression index scores among the mothers were comparable before and after the death, suggesting that changes in mother affiliative relationships were not a by-product of changes in overall interaction frequencies. Also, PI scores increased between the focal mother and three non-mothers after the death. Collectively, the shift in social dynamics between the focal mother and other group members after the infant death partially supported the mother-bonding hypothesis.

**Supplementary Information:**

The online version contains supplementary material available at 10.1007/s10329-022-00986-2.

## Introduction

Group living is suggested to have evolved when the benefits of increased sociality between conspecifics outweighed the resulting costs (Krause and Ruxton 2002). The benefits of group living include protection against predators (Hamilton 1971; Sorato et al. 2012), enhanced foraging success (Stander 1992), and improved access to mating partners, while the main cost of group living has been attributed to increased within-group competition for resources (van Schaik 1989; Sterck et al. 1997; Young and Isbell 2002). Relationships among members of a group are often differentiated, and a close and enduring relationship between two individuals is referred to as a bond (e.g., Cords 1997; Silk 2002). Forming and maintaining bonds with other individuals has been shown to enhance reproductive fitness, and is thus suggested to be adaptive for primates (Silk 2007; Ostner and Schülke 2014).

The strength of bonds is typically measured by rates of dyadic social behaviors, such as grooming and/or staying in close proximity (Cords 1997). Grooming is a common social behavior amongst primates. While it serves hygienic needs by removing ectoparasites from the skin, it is also used to establish and maintain social bonds (Dunbar 1991). In primates, evidence for a prominent social role of grooming besides its hygienic role includes the lack of a clear correlation between body size and grooming time, and a positive relationship between the time spent grooming and group size (Dunbar 1988, 1991). Proximity may be a less precise measure of social bonds than grooming because close proximity between individuals may be driven by other factors, such as attraction to a third party (Chapais 2001). However, staying in proximity may increase the opportunity for individuals to engage in affiliative behaviors, which in turn reflects the level of tolerance between two individuals and can inform us about the quality of the dyadic relationship.

In many group-living primates, strong social bonds are often observed among related individuals. According to Hamilton’s theory of kin selection, individuals can gain indirect fitness benefits by bonding with and acting prosocially towards their kin (Hamilton 1964). However, individuals may still form bonds with other unrelated individuals in the group through repeated, affiliative interactions over time, wherein the quality of past social interactions promotes future ones (Hinde 1976). The availability of related individuals within a group depends on the dispersal pattern of a given species. In most primate groups, males are the dispersing sex and females remain in their natal group. In these female-philopatric groups, females tend to form the strongest and most stable bonds with close female kin, as in the case of baboons (Silk 2003, 2009). Comparatively, female relationships appear to be more elusive in male-philopatric species due to the lack of close kin within the group (Nishida 1968; Slater et al. 2008; Williams et al. 2002; Wrangham et al. 1992; but see Langergraber et al. 2009; Lehmann and Boesch 2009; Rodseth and Novak 2006). Since female fitness is mostly constrained by access to food, relationships between unrelated females are largely determined by resource abundance and distribution (Sterck et al. 1997), whereby more abundant and evenly distributed resources reduce feeding competition and thus facilitate female sociality. While ecology may explain overall patterns of female sociality within an animal system, the potential factors that influence the tendency of females to associate and affiliate with particular females within the group are less clear.

Bonobos (*Pan paniscus*), together with chimpanzees (*Pan troglodytes*), are our closest living relatives, and live in male-philopatric societies (Kano 1982). Relative to chimpanzees, female-female relationships are particularly prominent in bonobo societies (Parish 1996). However, what drives female bondedness in bonobos remains a contentious topic. One of the putative explanations for female bonobo gregariousness is that the relatively stable and abundant food resources in bonobo habitat allow females to range in large mixed-sex parties without incurring high costs of feeding competition (Furuichi 2009). Even when food resources are limited, female bonobos have priority over males in feeding contexts, in which they defend and share food with other females (White and Wood 2007; Nurmi et al. 2018; Goldstone et al. 2016). Furthermore, female bonobos have relatively high social status within the group, which is acquired and maintained through female coalitions against male aggression (Surbeck and Hohmann 2013; Tokuyama and Furuichi 2016). Female sexual swellings are also thought to facilitate female association, affiliation, and socio-sexual behavior [i.e., genito-genital rubbing (Fruth and Hohmann 2006; Ryu et al. 2015; Surbeck et al. 2021)]. Another factor that may predict female social relationships in bonobos is maternal status. It has been shown that lactating female bonobos associate more frequently and spend more time in proximity with each other than with non-lactating females (Waller et al. 2011; Moscovice et al. 2017). This close association between lactating females may bring fitness benefits by providing a benign environment for females raise and socialize their offspring (Williams et al. 2002; Silk 2003).

In this paper, we aimed to investigate the tendency for females with infants (i.e., mothers) to affiliate with one another (i.e., mother-bonding hypothesis) in a group of wild bonobos at the Kokolopori Bonobo Reserve in the Democratic Republic of Congo. Specifically, we used the death of an infant as a natural experiment to investigate whether social relationships amongst female bonobos, particularly mothers, changed after one female lost her infant. If the formation of close female relationships in bonobos were related to the presence of similarly aged infants, we would expect the tendency to groom and stay in proximity between the female who has lost her infant (focal mother) and the other mothers to decrease after the death of the infant, as compared to before the death. We also documented changes in dyadic aggression frequencies around the death of the infant to exclude the hypothesis that changes in affiliation are a result of general changes in interaction frequencies. If female affiliative relationships changed after the infant death, we would expect changes in the proximity (PI) and grooming indices (GI), but not the aggression index (AI). While male–female relationships were not the focus of this study, we examined changes in female-male relationships around the death of an infant to explore potential social withdrawal from the focal mother associated with the infant death. Alternatively, as females can conceive again in a relatively short period of time following the death of an infant (Wallis 1997), males may be more attracted and increase their proximity to the focal mother after the infant death.

## Methods

### Study site and subjects

We collected data on the social behavior of a group of wild bonobos in the Kokolopori Bonobo Reserve, Democratic Republic of Congo, from August 2017 to July 2018. All the bonobos in the group, Ekalakala, have been habituated to human observers since 2016 (Surbeck et al. 2017). During the time of data collection, there was a total of 14 individuals in this group (three mature males, seven mature females, and four immature individuals). Despite its fission–fusion dynamics, this group was highly cohesive and most group members were observed during daily party (i.e., subgroup) follows. Our main study subjects were the seven mature females, two of which were sub-adults, and the three mature males (Table 1). There were three females with infants of similar age in the group: Peche, the female who lost her infant; Azur; and Violette. Before the infant death, Peche and Azur appeared to have a similar mothering style, and their infants were often seen socializing with other group members, whereas Violette had a more restrictive mothering style, and her infant was in body contact with her most of the time.

**Table 1:** Demographics of the Ekalakala group in the Kokolopori Bonobo Reserve, Democratic Republic of Congo, from August 2017 to July 2018

Individual	Sex	Birth year (estimated)	Age class	Offspring name	Offspring sex	Offspring birth year
Peche	Female	1992–2002	Adult	Prune	Female	2015
Azur	Female	199–2002	Adult	Acajou	Female	2014
Violette	Female	1982–1992	Adult	Vanille	Female	2016
Ivoire	Female	< 1982	Adult	Noir	Male	< 1982
Bleue	Female	< 1982	Adult	Brun	Male	2011
Eben	Female	2002–2007	Sub-adult	NA	NA	NA
Olive	Female	2002–2007	Sub-adult	NA	NA	NA
Gris	Male	1997–2002	Adult	NA	NA	NA
Noir	Male	< 1982	Adult	NA	NA	NA
Rouge	Male	1992–1997	Adult	NA	NA	NA

*NA* Not applicable

### The death of infant Prune

On 5 February 2018, Prune, the 3-year-old female infant of Peche was found dead just after Peche descended from a feeding tree. The cause of the infant’s death was unknown: there was no sign of injury on Prune or Peche, and there was no apparent aggression prior to the discovery of the dead infant. Peche carried Prune’s carcass for the rest of the day and up until the afternoon of 7 February. She was seen dragging, grooming, and inspecting the carcass, as well as removing and inspecting its intestines. Only the two sub-adult females in the group, Eben and Olive, approached and showed interest in the carcass (e.g., grooming the carcass and swatting flies away from it). On 7 February, the carcass was cannibalized by group members, including Peche. This type of cannibalistic behavior has been previously observed in this and another population of bonobos (Tokuyama et al. 2017).

### Data collection

All behavioral data were collected during daily party follows. We prioritized follows of the larger party whenever the group fissioned. During party follows, the accumulated party composition was recorded every 30 min. Grooming and proximity data were collected via scans conducted at 10-min intervals, during which the activity of every observed individual in the party was recorded, as well as the identity of the partner(s) that were in body contact with, within 1 m, and within 5 m of the observed individual. Agonistic interactions occurring within the followed party were recorded ad libitum throughout the day, with the identity of the aggressor and recipient, as well as the type of agonistic act (e.g., contact aggression, chase, charge, directed display), noted.

### Measures of dyadic social relationships

We compared all dyadic social relationships in the 6-month period before the death of Prune (BD) and the 6-month period after her death (AD). We adopted the sociality index developed by Moscovice et al. (2017) to quantify dyadic social relationships among all subjects by using grooming, proximity, and aggression measures. The GI, PI, and AI were all calculated separately for the purposes of this study because these three behavioral measures may reveal different information about the quality of social relationships in bonobos (Moscovice et al. 2017). The separate measures of GI, PI, and AI considered the observed rate of each behavior between each dyad, while taking into account total rates of that behavior by each of the dyads while both individuals were present in the party. The calculations of all the dyadic indices are as follows:

$$TBI_{xy} = \frac{{(X_{f} (XY_{tb} ) + Y_{f} (YX_{tb} ))}}{{(X_{f + tb} (Y_{pc} ) + Y_{f + tb} (X_{pc} ))}}$$

where the index of the target behavior (TBI), i.e., grooming, proximity, or aggression, of the dyad *XY* is calculated as the summation of the number of scans in which the individual *X* was performing the target behavior with individual *Y*, ($X_{f} (XY_{tb} )$), and the number of scans in which individual *Y* was performing the target behavior with individual *X*, ($Y_{f} (YX_{tb} )$). To account for the opportunity of each member of a dyad to perform the target behavior with the other member, as well as potential differences in the gregariousness of these individuals, we divided this summation by the total number of scans in which individual *X* was performing the target behavior with any individual while individual* Y* was present in the party ($X_{f + tb} (Y_{pc} )$) and vice versa ($Y_{f + tb} (X_{pc} )$). Thus, GI, PI, and AI can range from 0 to 1, with 0 representing individuals within the dyad who never engage in the target behavior with each other and 1 representing individuals within the dyad who only engage in the target behavior with each other, but not with other individuals. Because the behavioral scans and the party composition scans were recorded at different time intervals (i.e., grooming and proximity scans every 10 min, aggression whenever observed, and party composition every 30 min), we attached the party composition data that were recorded at the end of every 30 min to the data from the behavioral scans that were conducted within the respective 30-min time interval. For any missing party scans, we attached the data from the closest possible party scan to the data from the behavioral scan.

### Grooming indices

For the assessment of dyadic social relationships based on grooming interactions, we included behavioral scans in which the observed individual was in body contact with a partner and the behavior was recorded as “grooming.” We excluded from our analysis scans in which the identity of the partner could not be reliably recorded due to poor visibility. We were unable to account for grooming reciprocity and initiation as the beginning of grooming bouts was not always observed.

### Proximity indices

We considered two separate measures of proximity patterns, 1-m PI (PI_1m_) and 5-m PI (PI_5m_), as another way to quantify dyadic social relationships. When calculating the PI_1m_, we included scans in which the observed individual was within 1 m of other partner(s), and we excluded scans in which we could not reliably detect or identify the partner(s) due to poor visibility. When calculating the PI_5m_, we included scans in which the observed individual was within 5 m of other partner(s), excluding scans in which we could not reliably detect or identify the partner(s). We then examined whether the assessment of dyadic relationships varied substantially between PI_1m_ and PI_5m_ by investigating the correlation between the two indices. We found a moderate correlation between the two proximity measures (before death, *r* = 0.72, *P* < 0.001, *n* = 36; after death, *r* = 0.65, *P* < 0.001, *n* = 45). We also explored whether dyadic relationships differed when they were quantified using grooming interactions versus proximity patterns. To do that, we examined the correlation between GIs and each of the PIs. While neither the PI_1m_ nor PI_5m_ correlated significantly to the GI, the correlation between PI_1m_ and GI was stronger than that between PI_5m_ and GI, both before and after the death of Prune (before death PI_1m_, *r* = 0.25, *P* = 0.147, *n* = 36; after death PI_1m_, *r* = 0.02, *P* = 0.897, *n* = 45; before death PI_5m_, *r* = 0.22, *P* = 0.187, *n* = 36; after death PI_5m_, *r* = 0.00, *P* = 0.978, *n* = 45). Given the cohesive nature of the Ekalakala group and the less selective threshold of the 5-m proximity measure, the PI_1m_ is a better and more precise measure of dyadic social relationships than the PI_5m_. We therefore report results using the PI_1m_ throughout the main text and include results using the PI_5m_ in the Supplementary Information (Fig. S4). To keep all three behavioral indices (GI, PI, and AI) independent for analysis purposes, we only included proximity scans in which the activity of the observed individual was resting, playing, or feeding, and not grooming or agonistic.

### Aggression indices

For the calculation of AIs, we included all observations of directed agonistic acts (see above) between our subjects, and assessed the AI for each dyad using the same equation applied for GI and PI. Due to the way the behavioral scan data were collected, there were likely unrecorded grooming bouts and aggressions, as well as changes in the proximity of individuals that occurred between two 10-min scans.

To test the mother-bonding hypothesis and investigate whether the death of Prune influenced the dyadic relationships between Peche, the mother of Prune, and other mothers in the group, we compared behavioral index scores for dyads between Peche and mothers, Peche and non-mother females, and Peche and males, before and after the infant’s death.

### The immigration of new female Olive

One of the sub-adults, Olive, was a new female who immigrated into the group in the middle of the study period, on 16 December 2017. Given that the immigration occurred around the time that Prune died, we included all social interactions between Olive and our study subjects when calculating the GI, PI, and AI in the period after the death. Behavioral observations of Olive were sparse in the period before the death, possibly due to her habituation level at that time. Thus, we did not consider Olive in our analyses for the period before Prune’s death. While we were unable to account for potential effect of Olive’s immigration on dyadic relationships in the group after the death, we report behavioral index scores for all dyads (including dyads not involving Peche, as well as dyads of the new immigrant female Olive in the after death period) in the Supplementary Information, and discuss any changes in overall social relationships that may be related to the immigration of Olive.

## Results

### Behavioral index scores for all dyads before versus after the death

The GI scores for all dyads ranged from 0.000 to 0.579 (mean ± SD = 0.154 ± 0.153) in the period before the death, and from 0.000 to 0.653 (mean ± SD = 0.142 ± 0.133) in the period after the death. The PI_1m_ scores for all dyads ranged from 0.033 to 0.434 (mean ± SD = 0.190 ± 0.101) in the period before the death, and from 0.000 to 0.448 (mean ± SD = 0.235 ± 0.127) in the period after the death. The AI scores for all dyads ranged from 0.000 to 0.939 (mean ± SD = 0.123 ± 0.195) in the period before the death, and from 0.000 to 0.444 in the period after the death (mean ± SD = 0.124 ± 0.129).

### GI scores for dyads of Peche before and after the death

Overall, GI scores for dyads of Peche and females were lower in the period after the death (mean ± SD = 0.171 ± 0.085) than in the period before the death (mean ± SD = 0.270 ± 0.209). Similarly, GI scores for dyads of Peche and males were lower in the period after the death (mean ± SD = 0.016 ± 0.022) than before the death (before death, mean ± SD = 0.032 ± 0.013).

**Fig. 1:**
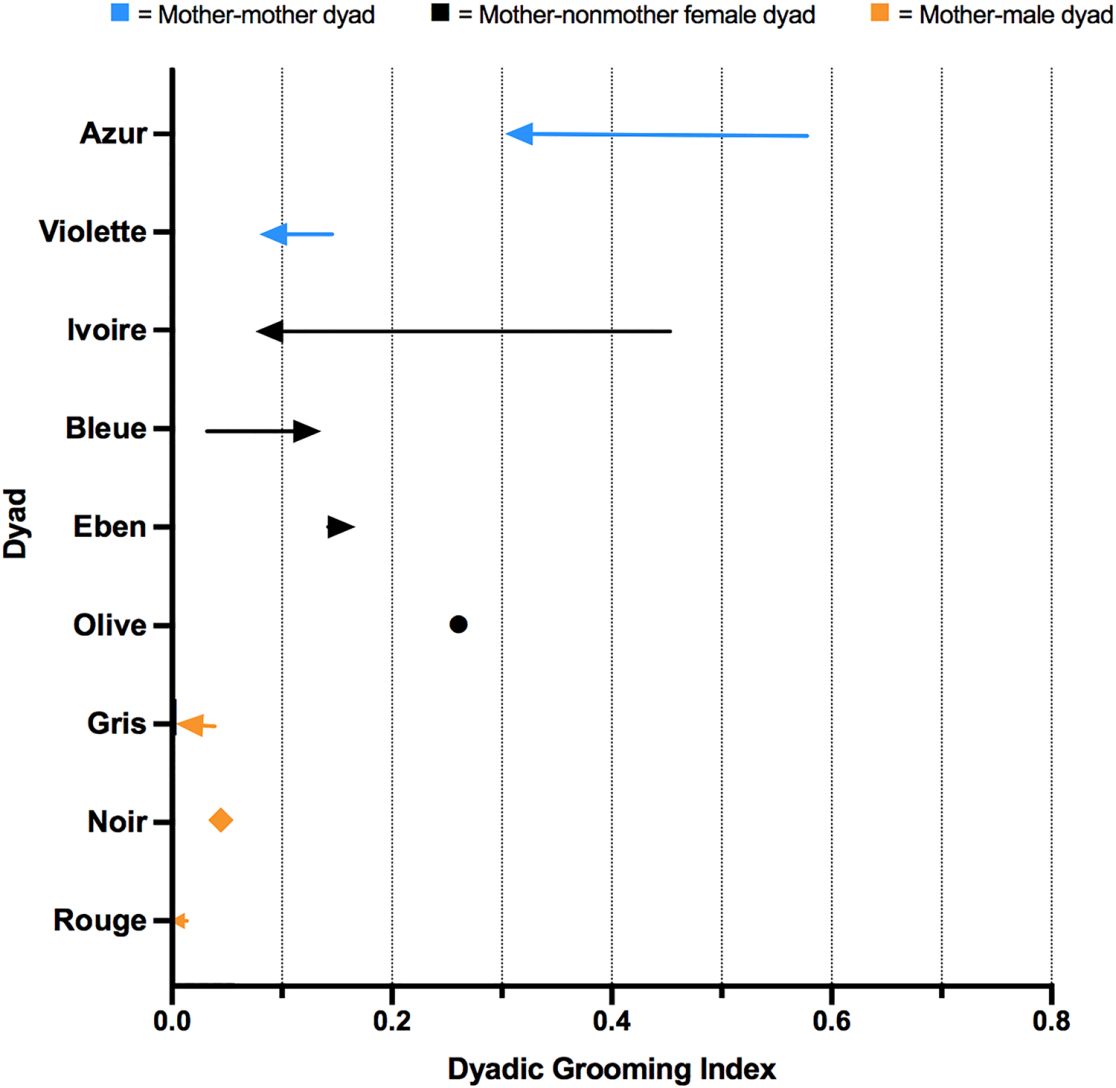
Changes in grooming index (GI) scores for dyads of Peche from the period 1 August 2017 to 4 February 2018 (before death; BD) to the period 5 February to 31 July 2018 (after death; AD).* Arrows* indicate the directional changes in dyadic scores for Peche and another individual from the BD to AD period. The* diamond* indicates a change in the dyadic score of less than 0.01, and the* circle* indicates the dyad score for Peche and Olive in the AD period. Figure produced in Graph Pad Prism, version 9.0.0

In the 6 months before the death of Prune, one of the mother dyads, Peche and Azur, had the highest GI score among all dyads of the group (GI_BD_ = 0.579; Fig. 1; Fig. S1). In the period after the death, Peche and Azur still groomed each other more than Peche groomed any of the other individuals (GI_AD_ = 0.305; Fig. 1; Fig. S1), but their GI score was much lower after than before the death (∆GI from BD to AD = − 0.274; Fig. 1). The GI score for another mother dyad, Peche and Violette, also decreased in the period after the death (∆GI from BD to AD = − 0.064), although the decrease was less substantial than that observed between Peche and Azur (Fig. 1). There was a drastic decrease in the GI score for Peche and a non-mother, Ivoire, after the death (∆GI from BD to AD = − 0.371; Fig. 1). After the death, Peche and the new female, Olive, developed a strong grooming relationship that was comparable to that between Peche and Azur (Peche and Olive, GI_AD_ = 0.261; Fig. 1). The GI score for the third mother dyad, Azur and Violette, was relatively low before the death of Peche’s infant (GI_BD_ = 0.019; Fig. S1), but it increased moderately in the period after the death (∆GI from BD to AD = 0.168; Fig. S1).

### PI_1m_ scores for dyads of Peche before and after the death

The PI scores for the dyads of Peche and females were slightly lower in the period after the death (mean ± SD = 0.233 ± 0.110) than before the death (mean ± SD = 0.247 ± 0.088). PI scores for dyads of Peche and males were generally higher after (mean ± SD = 0.256 ± 0.083) than before the death (mean ± SD = 0.165 ± 0.071).

**Fig. 2:**
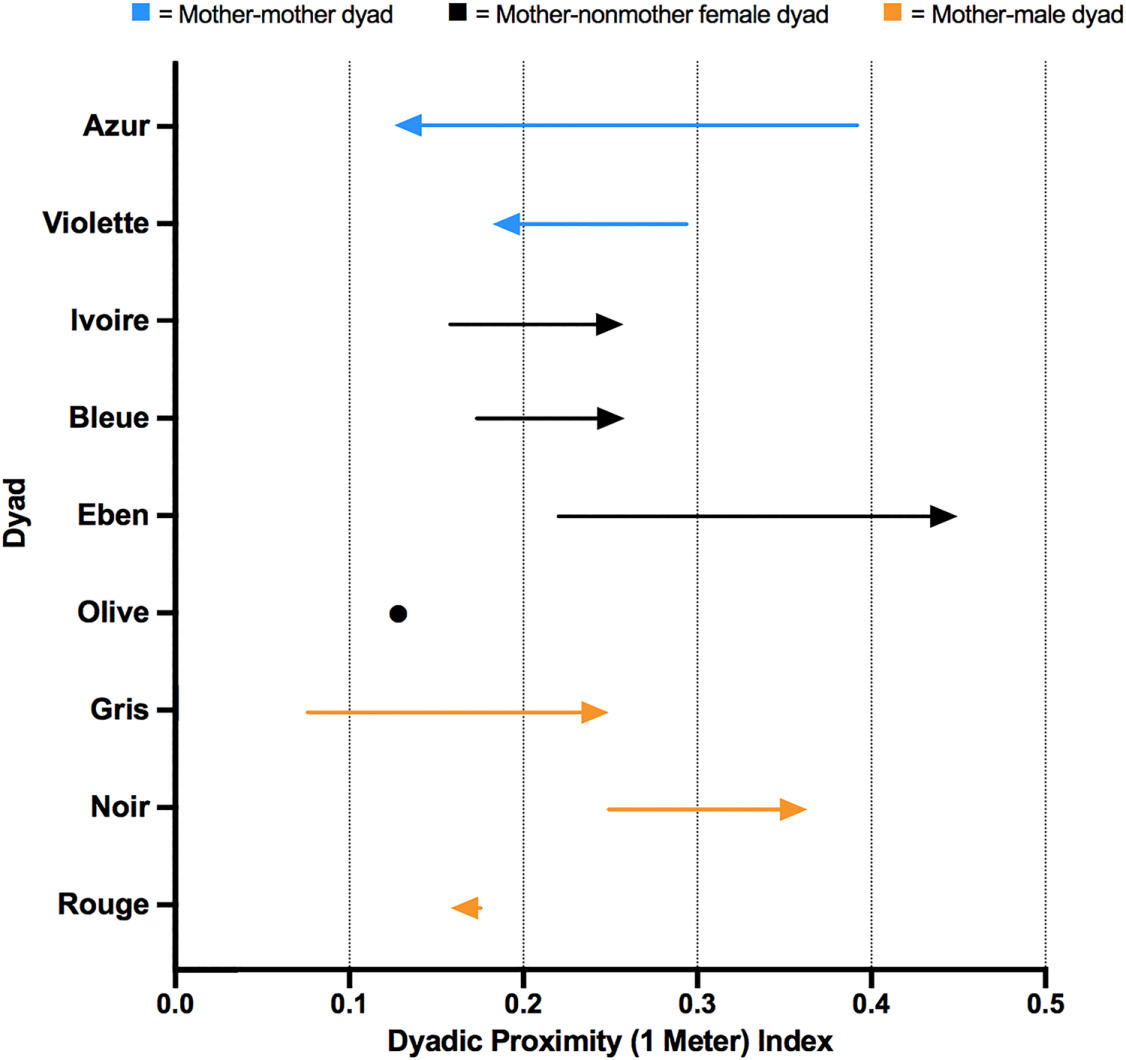
Changes in proximity index (PI) scores for dyads of Peche from the period 1 August 2017 to 4 February 2018 (BD) to the period 5 February to 31 July 2018 (AD).* Arrows* indicate the directional changes in dyadic scores for Peche and another individual from the BD to AD period. The* circle* indicates the dyad score for Peche and Olive in the AD period. Figure produced in Graph Pad Prism, version 9.0.0. For other abbreviations, see Fig. 1

In the period before the death, Peche and Azur had the highest PI scores with each other over those of Peche with any of the other individuals (PI_BD_ = 0.393; Fig. 2; Fig. S2). However, this was not the case in the period after death, as the PI score for Peche and Azur decreased during this period (PI_AD_ = 0.125). While the PI score for Peche and Violette also decreased in the period after the death, as opposed to before the death (PI_BD_ = 0.295; PI_AD_ = 0.184), the PI scores for Peche and all non-mother females (i.e., Ivoire, Bleue, and Eben) increased substantially after the death (Fig. 2). The PI score for the mother dyad of Azur and Violette was higher in the period after the death when compared to before the death (PI_BD_ = 0.101; PI_AD_ = 0.275; Fig. S2).

### AI scores for dyads of Peche before and after the death

AI scores for dyads of Peche and females were slightly lower in the period after the death (mean ± SD = 0.052 ± 0.073) than before the death (mean ± SD = 0.074 ± 0.130), whereas AI scores for dyads of Peche and males were slightly higher after than before the death (BD, mean ± SD = 0.035 ± 0.027; AD, mean ± SD = 0.049 ± 0.041).

**Fig. 3:**
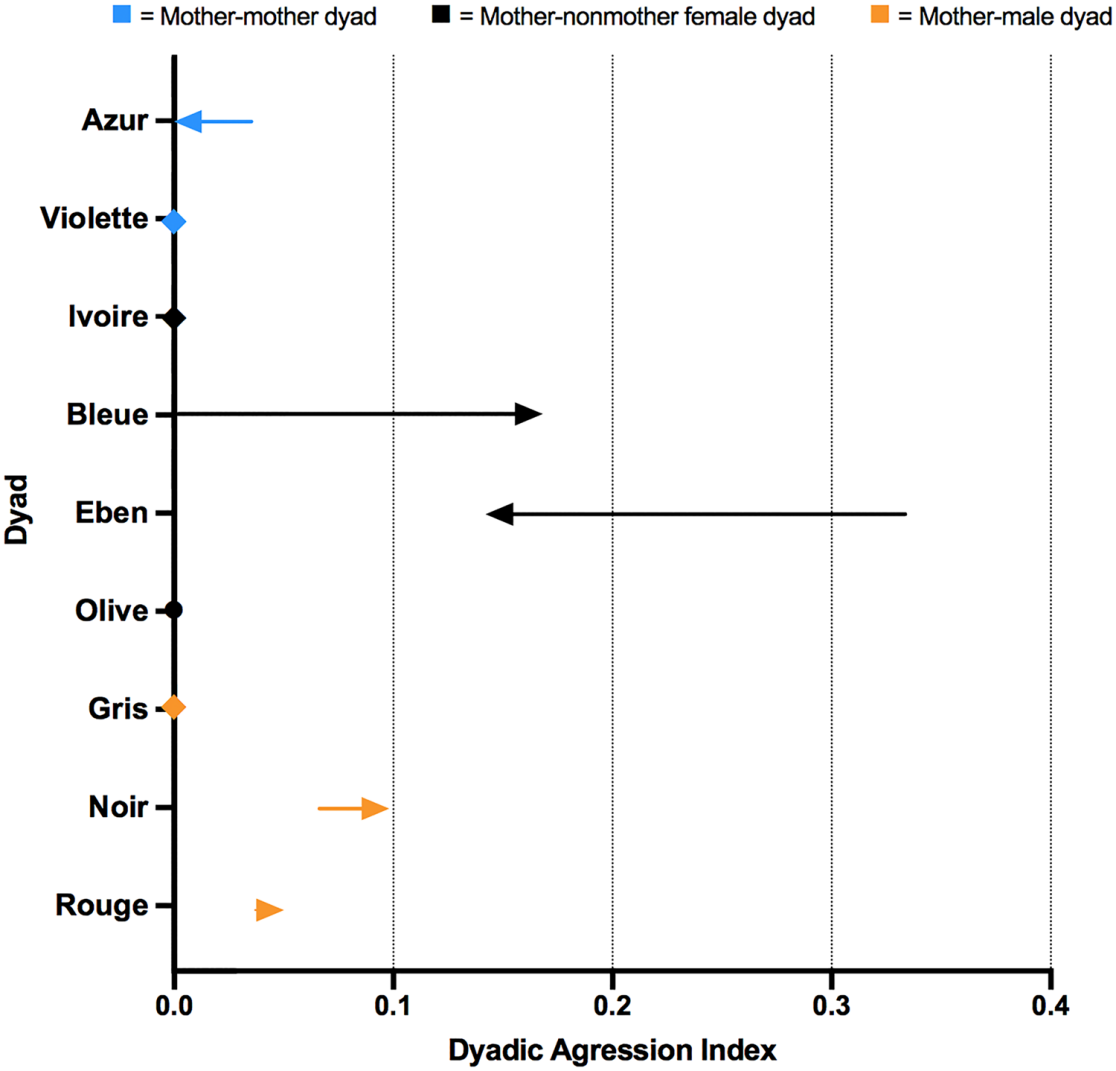
Changes in aggression index (AI) scores for dyads of Peche from the period 1 August 2017 to 4 February 2018 (BD) to the period 5 February to 31 July 2018 (AD).* Arrows* indicate the directional changes in dyadic scores for Peche and another individual from the BD to AD period.* Diamonds* indicate no change in dyadic score, and the* circle* indicates the dyad score for Peche and Olive in the AD period. Figure produced in Graph Pad Prism, version 9.0.0. For other abbreviations, see Fig. 1

In the period before the death, the highest AI score among all dyads of Peche was between Peche and Eben (AI_BD_ = 0.333; Fig. 3). However, their index score decreased considerably in the period after the death (AI_AD_ = 0.143), whereas the AI score for Peche and Bleue increased substantially (AI_BD_ = 0.000; AI_AD_ = 0.167). There was relatively little aggression between Peche and Azur before the death (AI_BD_ = 0.036), and there was no aggression between these two mothers after the death (AI_AD_ = 0.000). There was also no aggression observed between Peche and Violette, Peche and two non-mothers, Ivoire and Olive, or between Peche and one of the males, Gris, throughout the study period (Fig. 3). The AI scores for Peche and the other males, and for the remaining mother dyad, Azur and Violette, were all comparable before and after the death (Fig. S3).

## Discussion

The goal of this study was to test the mother-bonding hypothesis by investigating how the death of an infant influenced the mother’s social relationships within a group of wild bonobos. We found partial support for the mother-bonding hypothesis. When inspecting the dyadic relationships among mothers in greater detail, we observed some changes in the strength of these relationships before and after the death. First, the affiliative relationship between two of the mothers, Peche, who lost her infant, and Azur, who had a similarly aged infant, was weaker after than before the death of Peche’s infant, as indicated by substantially lower GI and PI scores after than before death (Figs. 1, 2). Second, the affiliative relationship between Peche and another mother, Violette, was also weaker after the infant death, though they did not have a strong affiliative relationship before the death (Fig. 1). Third, while the relationship within the remaining mother dyad, Azur and Violette, was not particularly strong before the infant death, their relationship was strengthened after the death, as shown by an increase in GI and PI scores (Figs. S1, S2). Below, we discuss the behavioral strategies of mothers, and interpret changes in social relationships among mothers, as well as non-mothers between the before and after death periods, in relation to predictions of the mother-bonding hypothesis.

The mother-bonding hypothesis predicts that mothers of similarly aged infants tend to have stronger relationships with each other than with other individuals in the group. Accordingly, the three mothers, Peche, Azur, and Violette, should have had the strongest relationships among all the female dyads before the death of Prune, and the relationship between Peche and the other two mothers should have been weaker after the death of Peche’s infant, whereas the relationship between Azur and Violette should have remained strong. In line with the mother-bonding hypothesis, the strongest female-female relationship before the death was in a mother dyad, that of Peche and Azur. Even though the relationship between these two mothers was substantially weakened after the death, it was still one of the stronger relationships among all the female dyads, as indicated by its relatively high dyadic grooming score (Fig. 1). Regardless of the presence of infants, the similar ages of Peche and Azur may have affected their social preference for each other, as is the case for similarly aged female baboons (Silk et al. 2006). Also, past, positive interactions between Peche and Azur may have motivated the dyad to continue to socialize even after the infant death (Hinde 1976).

The patterns of relationships between the other mother dyads (i.e., Peche and Violette, Azur and Violette) seem inconsistent with the mother-bonding hypothesis. These mothers did not have particularly strong social relationships in either the before death or the after death period. This may be attributed to differences in individual personality and/or mothering style. During the study period, Violette restricted her infant’s movement and was always in body contact with the infant. Thus, it was difficult for other group members to socialize with Violette’s infant even when they were in close proximity. Under the assumption that mothers form strong bonds for the social benefits of their infants (Williams et al. 2002; Silk 2003), it was not surprising to see relatively weak relationships between Violette and the other mothers, despite the presence of similarly aged infants.

There were also substantial changes in the dyadic relationships between Peche and non-mothers Ivoire, Bleue, and Eben. While all these relationships were strengthened through proximity after compared to before the death (Fig. 1), there seemed to be greater tolerance between Peche and Eben, but a lower tolerance between Peche and Bleue, as suggested by the changes in their dyadic AI scores (Fig. 2). Although these changes seem unrelated to the mother-bonding hypothesis, they indicate that female social relationships are dynamic and flexible in bonobos (see also Moscovice et al. 2017). The variation in female dyadic AIs may also reflect changes in female-female competition within the group. Soon after the death of her infant, Peche was cycling again and had a new infant 9 months after Prune’s death. Given that the length of gestation in bonobos is around 229–242 days (Hashimoto et al. 2022; Heistermann et al. 1996), Peche likely conceived around 1 month after Prune’s death. The changes in her reproductive state may have impacted her social relationships with males. While her grooming relationships with all males remained similar throughout the study period, her proximity relationships with Noir (the highest ranking male) and Gris (the lowest ranking male) were stronger after than before the death. It is possible that these males were attracted to Peche due to her return to fertility after her infant’s death and thus increased their proximity to her to gain mating opportunities. Furthermore, males may have remained close to Peche during pregnancy and lactation to improve their chance of siring her next offspring, a strategy that has been shown in male olive baboons (Städele et al. 2019).

One major confounding variable that we were unable to account for in this study was the potential impact of the new immigrant female, Olive, on female sociality. In female-dispersal primate species, female immigration can influence social relationships within the group, especially those among females (Idani 1991; Kahlenberg et al. 2008; Nishida 1989; Sakamaki et al. 2015; Toda and Furuichi 2020, 2022). For example in bonobos, new immigrant females tend to receive less aggression from and groom more often with high-ranking, senior females than other females in the group, suggesting that high-ranking, senior females may be more tolerant and attractive social partners, especially for new immigrant females (Toda and Furuichi 2020, 2022). However, since we did not set up the study to specifically test the mother-bonding hypothesis or the impact of new immigrant females on female sociality, we were unable to disentangle the effect of the infant death from that of Olive’s immigration in our analyses.

**Fig. 4:**
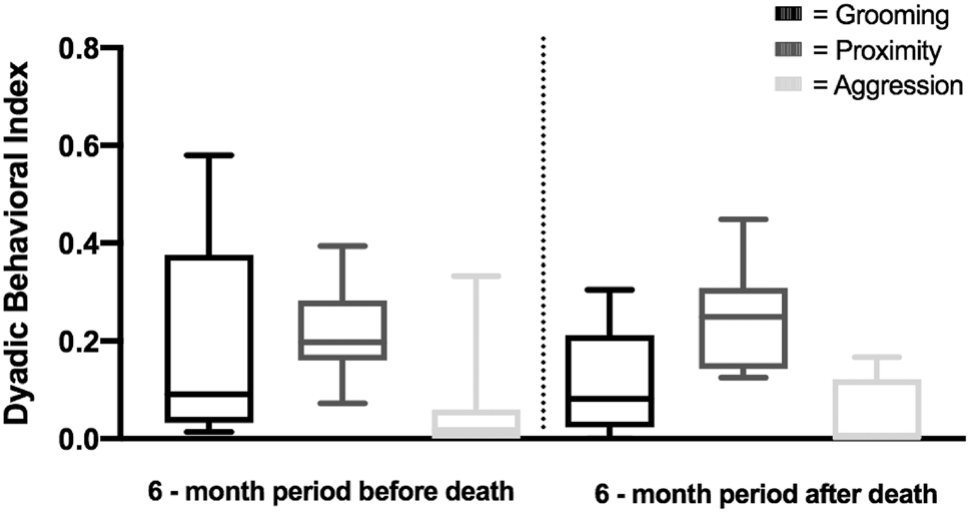
Box-and-whisker plot showing the GI, PI, and AI scores for dyads of Peche in the BD and AD periods. Shown are the median dyadic scores *(middle horizontal lines), quartiles (boxes), as well as 2.5 and 97.5 percentiles (error bars)* for the respective time period. Figure produced in Graph Pad Prism, version 9.0.0. For abbreviations, see Figs. 1, 2, and  3

Although there was an overall decrease in Peche’s grooming scores with other individuals after the death of her infant, her proximity scores were higher and her aggression scores were similar after as compared to before the death. This indicates that changes in her dyadic relationships were not a result of her social withdrawal (see Fig. 4). Our data also suggest that the two socio-positive measures, grooming and proximity, may reveal different aspects of female affiliative relationships. When assessing the overall group patterns, we found that the GIs amongst all dyads were far more differentiated than the PIs, given that the variation of dyadic index scores was larger in the former than the latter, especially in the period before the death (see Fig. 4). The same pattern was described in another bonobo population, LuiKotale (Moscovice et al. 2017). In addition, the small variation in PI scores across dyads may reflect the cohesiveness of the Ekalakala group. When observers followed a party of Ekalakala throughout the day in another study (Lucchesi et al. 2021), most group members were present and often remained in proximity to each other (91% of the group members were observed, on average, per observation day). On the other hand, grooming likely requires greater agency from partners than tolerating the close proximity of another individual does, as grooming is associated with higher opportunity costs than proximity (Dunbar 2010). GIs thus seem to be a better measure than PIs in revealing differentiated social relationships, especially in cohesive groups like Ekalakala.

The tendency for females with infants to affiliate with one another is not limited to species that exhibit particularly strong and enduring female-female relationships. Lactating spider monkeys (*Ateles geoffroyi*), for example, display greater association with one another (Shimooka 2015; Slater et al. 2007) even though female-female relationships are generally weak in this species (Aureli and Schaffner 2008). Social integration, which can be achieved through strong dyadic relationships, provides females with a broad range of direct and indirect fitness benefits. In species such as bonobos and chimpanzees, where social relationships are well-differentiated within the group, association between unrelated females may vary with the presence and sex of offspring [bonobos (Waller et al. 2011; Moscovice et al. 2017); chimpanzees (Foerster et al. 2015)]. In particular, females with infants may benefit from remaining in close proximity to one another as this provides opportunities for their infants to socialize with peers (Williams et al. 2002), increases predator protection for infants by creating a physical barrier (Silk et al. 2009; Cheney et al. 2006), and shields infants from within-group aggression, allowing them to feed safely (Silk 2003). In both chimpanzees and bonobos, females continue to exert a strong influence on the fitness of their male (philopatric) offspring even during offspring adulthood, such as enhancing their dominance status, as well as reproductive opportunities and success of their offspring (Crockford et al. 2020; Surbeck et al. 2011, 2019). In the present study, Peche’s infant was a female, and her social strategies may have been different if her infant had been a male. A promising avenue of future research would be to investigate whether the sex of the offspring impacts mother social relationships differently.

It is apparent from our data and previous studies that mother bondedness is not the only mechanism underlying female sociality. In bonobos, female sexual swellings are signals that attract males as mating partners and females as social partners (Ryu et al. 2015; Surbeck et al. 2021). In other species, friendly relationships among females increase co-feeding tolerance and resource-sharing opportunities, thus improving access to resources (Tiddi et al. 2011; Samuni et al. 2018). Furthermore, grooming among females, as well as among males, has been shown to reduce baseline glucocorticoid levels, which may be particularly beneficial after a stressful event, such as infanticide or the loss of a close partner (Engh et al. 2006; Wittig et al. 2016). Additionally, unrelated females that groom more often also tend to support each other in coalitions (Seyfarth and Cheney 2012; but see Tokuyama and Furuichi 2016). Therefore, females may form strong bonds not only for the survival and well-being of their infants, but also for their own health and fitness. Further investigation into potential social strategies of females with and without infants in other male-philopatric species will help improve our understanding of how social relationships are formed and maintained among unrelated females.

## Supplementary Information

Below is the link to the electronic supplementary material.

Supplementary file1 (DOCX 1343 KB)
