# Supplementary material for: Mothers stick together: how the death of an infant affects female social relationships in a group of wild bonobos (Pan paniscus)
Source: Primates. 2022 Apr 18;63(4):343–53. doi: 10.1007/s10329-022-00986-2 (PMC9273548; doi:10.1007/s10329-022-00986-2)
Supplement: Supplementary file 1 — Supplementary file1 (DOCX 1343 KB) [file 10329_2022_986_MOESM1_ESM.docx]

**SUPPLEMENTARY INFORMATION**

**Title:** Mothers stick together: How the death of an infant affects female social

relationships in a group of wild bonobos (*Pan paniscus*)

**Authors:** Leveda Cheng^a,b,*^, Amber Shaw^a,*^, Martin Surbeck^a,b^

^a^Department of Human Evolutionary Biology, Harvard University, 11 Divinity Ave, Cambridge, MA 02138, USA

^b^Max Planck Institute for Evolutionary Anthropology, Deutscher Platz 6, 04103 Leipzig, Germany

^*^Leveda Cheng and Amber Shaw contributed equally to this study.

**Correspondence:** L. Cheng

levedacheng@g.harvard.edu

Department of Human Evolutionary Biology, Harvard University, 11 Divinity Ave, Cambridge, MA 02138, USA

*Supplementary figures*


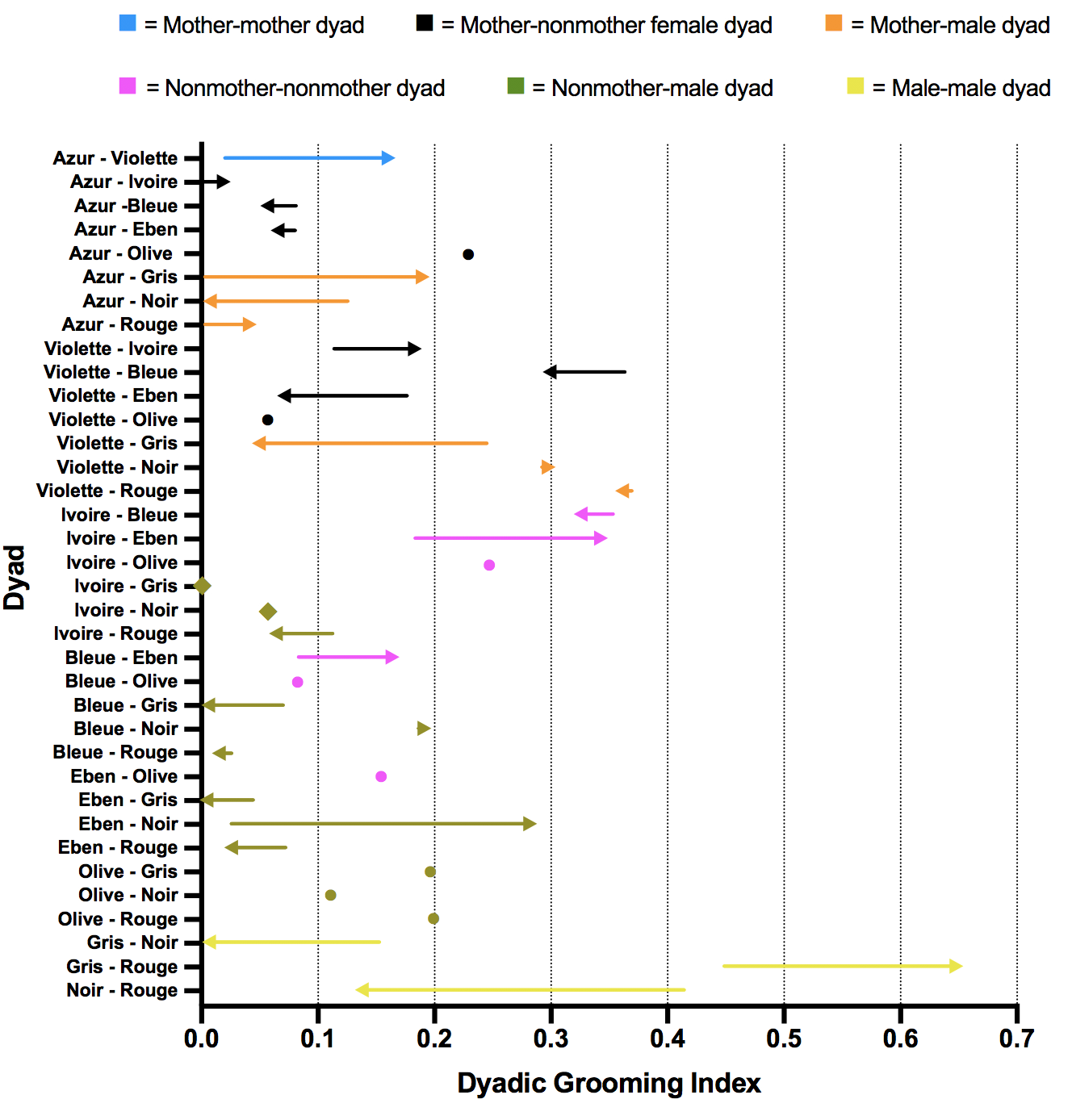


**Fig. S1.** Changes in grooming index scores for all dyads (except dyads of Peche) from the period August 1st 2017 – February 4th 2018 (before death; BD) to the period February 5th 2018 – July 31st 2018 (after death; AD). Arrows indicate the directional changes in dyadic index scores from the BD to AD period. Diamonds indicates change in dyadic scores less than 0.01 and circles depicts the dyad score for Peche and Olive in the AD period.


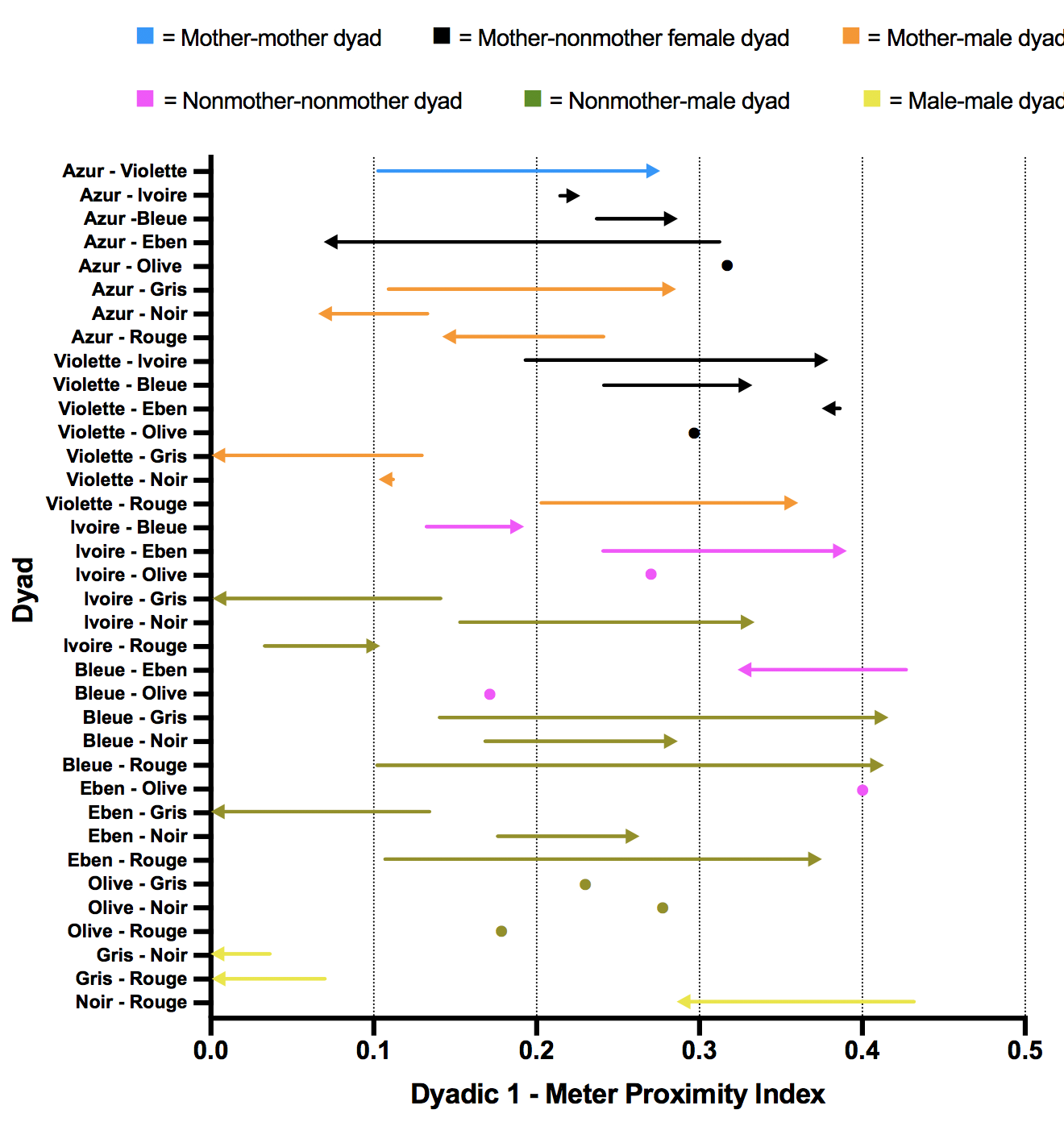


**Fig. S2.** Changes in 1-meter proximity index scores for all dyads (except dyads of Peche) from the period August 1st 2017 – February 4th 2018 (before death; BD) to the period February 5th 2018 – July 31st 2018 (after death; AD). Arrows indicate the directional changes in dyadic index scores from the BD to AD period. Circles depict the dyads of Olive in the AD period.

**
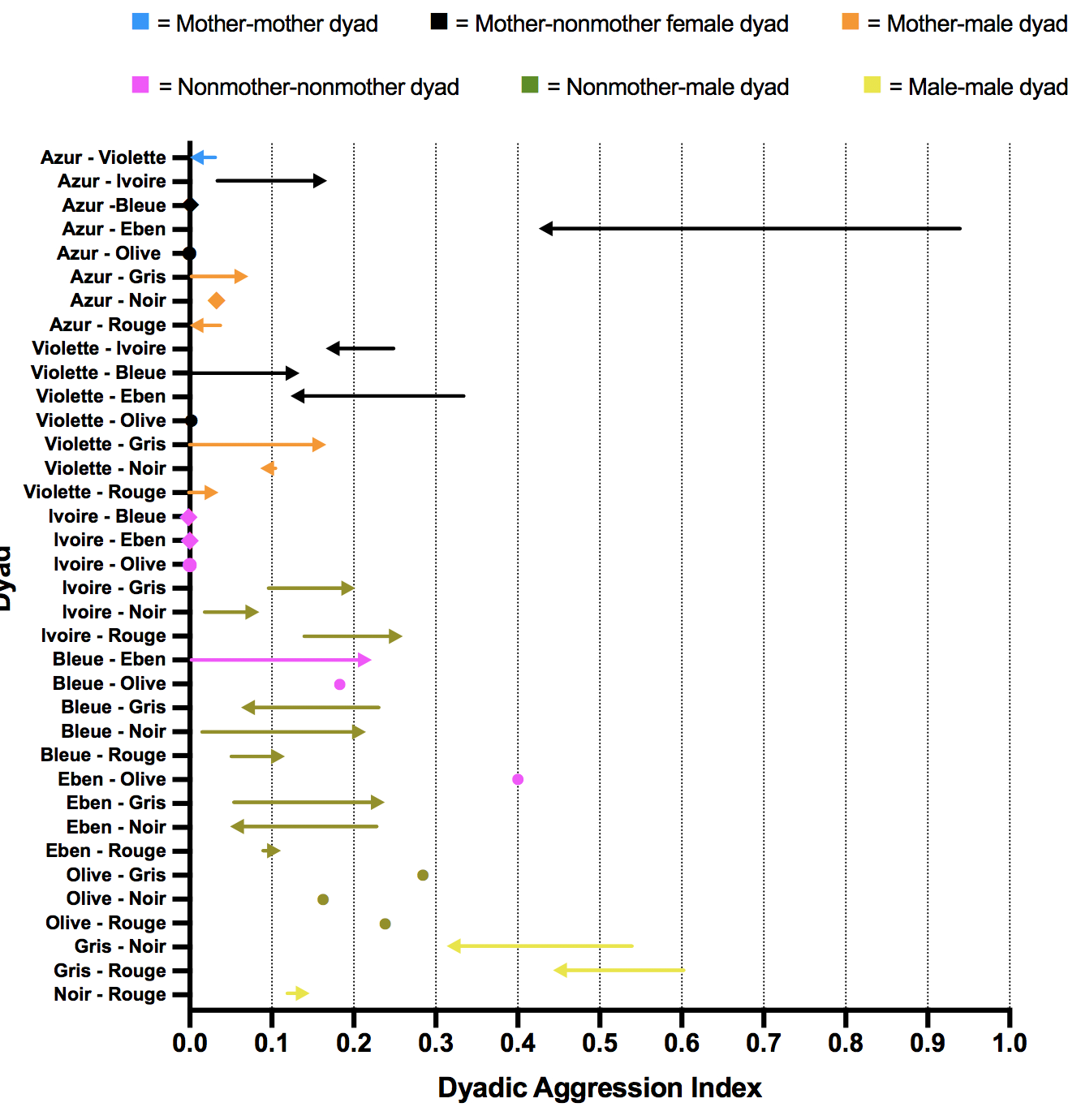
**

**Fig. S3.** Changes in aggression index scores for all dyads (except dyads of Peche) from the period August 1st 2017 – February 4th 2018 (before death; BD) to the period February 5th 2018 – July 31st 2018 (after death; AD). Arrows indicate the directional changes in dyadic index scores from the BD to AD period. Diamonds indicate no change in dyadic index scores and circles depict the dyads of Olive in the AD period.


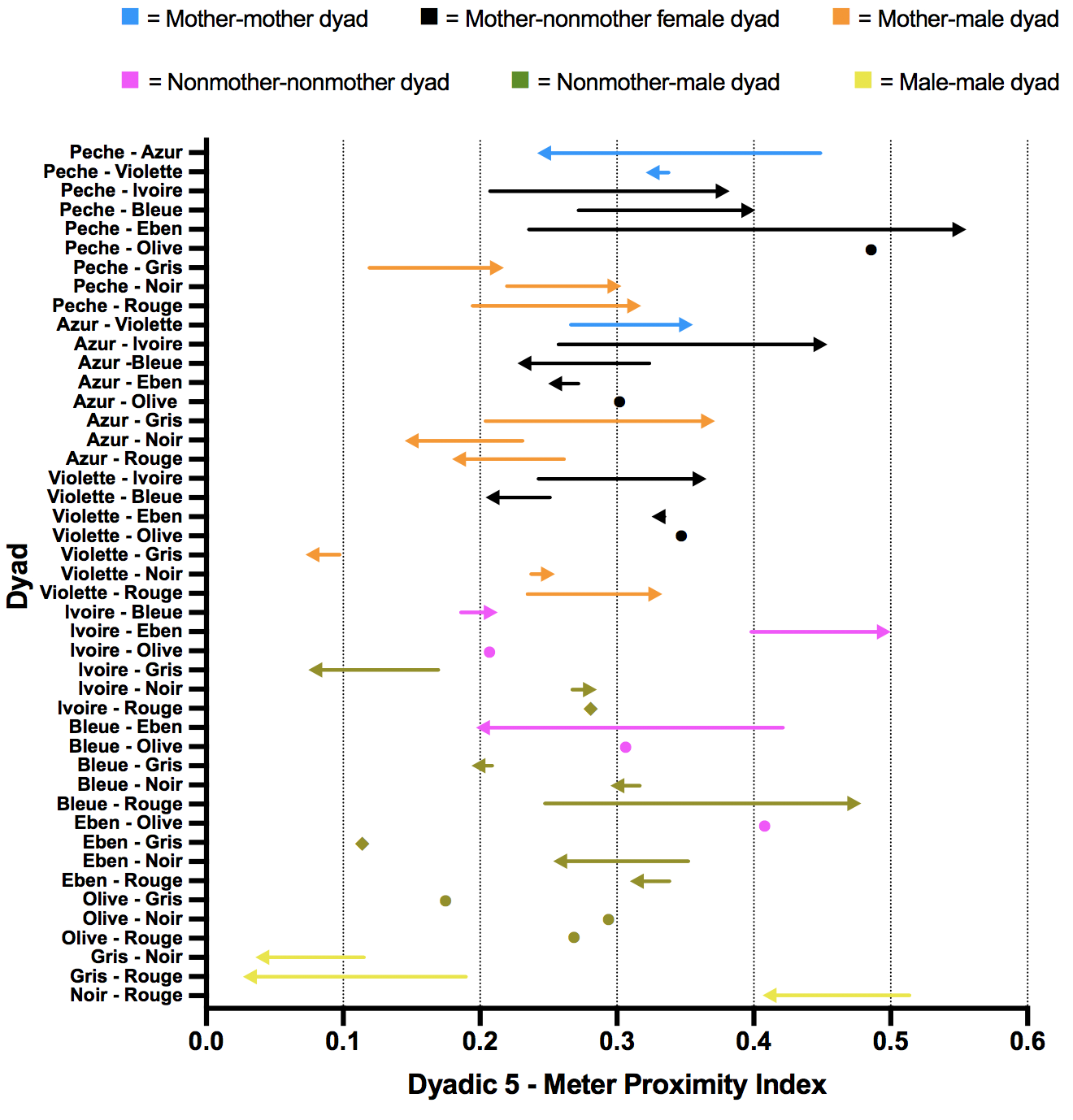


**Fig. S4.** Changes in 5-meter proximity index scores for all dyads from the period August 1st 2017 – February 4th 2018 (before death; BD) to the period February 5th 2018 – July 31st 2018 (after death; AD). Arrows indicate the directional changes in dyadic index scores from the BD to AD period. Diamonds indicate no change in dyadic index scores and circles depict the dyads of Olive in the AD period.
